# Data on biodistribution and dose calculation of ^99m^Technetium -Dimercaptosuccinic acid in pediatric patients using a hybrid planar/single emission computed tomography method

**DOI:** 10.1016/j.dib.2020.106232

**Published:** 2020-08-29

**Authors:** Mahmoud Bagheri, Masoumeh DorriGiv, Marjaneh Hejazi, Mohammad Reza Fouladi, Ali Asghar Parach

**Affiliations:** a Research Center for Molecular and Cellular Imaging, Tehran University of Medical Sciences, Tehran, Iran; b Department of Medical Physics and Biomedical Engineering, Faculty of Medicine, Tehran University of Medical Sciences, Sina Campus, Tehran, Iran; c Nuclear Medicine Research Center, Department of Nuclear Medicine, Ghaem Hospital, Mashhad University of Medical Sciences, Mashhad, Iran; d Department of Medical Physics, Shahid Sadoughi University of Medical Sciences, Yazd, Iran

**Keywords:** Radiation absorbed dose, Biodistribution, ^99m^Tc-DMSA, Planar/SPECT method

## Abstract

The Biodistribution and absorbed dose data from the administration of radiopharmaceuticals are necessary to analyze the risk-benefit of the procedure. It has particular significance in children, as their metabolism is very different from adults. ^99m^Tc-DMSA scintigraphy is the golden standard imaging technique for the assessment of renal involvement in febrile urinary tract infection and renal sequels. However, ^99m^Tc-DMSA biodistribution data for children are scarce and usually outdated which have been obtained by older methods. In this data article, we analysed the biodistribution of ^99m^Tc-DMSA in 12 pediatric patients using planar/SPECT method. In addition, the radiation absorbed doses were calculated by MIRDOSE software.

## Specifications Table

**Table:** 

Subject	Nuclear medicine, clinical research
Specific subject area	Biodistribution analysis and absorbed dose calculation of ^99m^Tc-DMSA (Technetium-99m-dimercaptosuccinic acid) in pediatric patients.
Type of data	Tables, Figures
How data were acquired	Direct collection of tissues from pediatric patients at different time-points using planar, SPECT (single emission computed tomography), and MRI (magnetic resonance imaging).
Data format	Raw, Analyzed.
Parameters for data collection	Each patient underwent 3 to 5 planar scans, and also single SPECT scan after ^99m^Tc-DMSA injection with a dual-head gamma camera system (a parallel hole and LEHR [low energy high resolution] collimator). In addition, each patient imaged by MRI before injection.
Description of data collection	All acquisition data were stored on the computer, including count-rates and measurement times. For all images, the count-rates were determined using suitable ROIs (region of interests), as well as a region surrounding each ROI was used for background correction. The cumulative activity and residence times for each source organ were calculated from count-rates with planar/SPECT method.
Data source location	Shahid Sadoughi Hospital of Yazd, Iran
Data accessibility	Raw and processed data are available with the article.

## Value of the Data

• These data present the biodistribution and absorbed dose of ^99m^Tc-DMSA for children as sensitive to ionizing radiation.
• Our data provide important information on the value of hybrid planar/SPECT and MRI techniques for biodistribution measurement and will be useful to calculate in absorbed dose more accurately.
• The date can be used for children patients in renal scintigraphy with ^99m^Tc-DMSA for the best/optimize time during the imaging process especially in busy nuclear medicine departments.
• These data will be of interest to all those scientists who have access the biodistribution and absorbed dose data from the administration of radiopharmaceuticals which are necessary to analyze the risk-benefit of the procedure.
• The data can be used to further improve the standardization of children's dosimetric assessments and recommendations for activity administration for future studies on risk prognostication in clinical practice.

## 1 Data Description

In previous data, for calculating the absorbed dose of ^99m^Tc-DMSA in pediatric, a planar method was used [1] at a short time period acquisition after injection [2]. Furthermore, in the past researches, the lateral planar images have used to obtain the organ and patient body thicknesses for self-attenuation and background corrections, and also transmission factor [2]. However, we used MRI method to calculate these corrections which is more accurate than the planar method as well as without unnecessarily patient's absorbed dose compared to computed tomography (CT) images [3].

In this data article, the aim was to obtain biodistribution data with planar/SPECT method [3] from children at various ages and degrees of renal dysfunction after the administration of ^99m^Tc-DMSA in order to look for evidence of age-dependency. Herein we have provided the biodistribution in different time periods ranging from 30 min to 19 h. In addition, the percentage of ^99m^Tc-DMSA uptake in source organs and reminders are separately calculated for each patient.

## 2 Experimental design, materials and methods

### 2.1 Patient studies

Twelve pediatric patients including 4 males and 8 females, aged from 3 to 12 years old have participated in this data article. Informed consent was obtained from all participants after the procedures were fully explained and the study was approved by Shahid Sadoughi University of Medical Sciences (Yazd, Iran) with the registration number of “4137″. The patients had the genitourinary abnormalities problem. They were injected with 86–170 MBq with the mean value ± standard deviation of 116.7 ± 26.7, ^99m^Tc-DMSA for acquisition Nuclear Medicine imaging. The patients’ demography, including height, weights, and ages has been shown in Table 1.

**Table 1:** Demographic data for the patients and administered activity to each patient are also included.

Patient number	Age (yr)	Sex	Weight (kg)	Height (cm)	Administered activity (MBq)
1	7	F	23	112	98
2	5	M	19	119	115
3	8	M	25	130	107
4	5	F	20	110	106
5	7	F	20	114	170
6	4	F	18	105	102
7	12	F	43	145	169
8	3	F	15	98	115
9	7	F	21	108	86
10	4	F	13	100	127
11	4	M	15	100	107
12	4	M	14	106	98

### 2.2 Imaging procedures

#### 2.2.1 Planar and SPECT images

The injection activity measurements were obtained using a calibrated ‘dose calibrator’ (Capintec, Inc., Ramsey, New Jersey, USA). A dual-head gamma camera system (Philips ADAC, forte) with a parallel hole, LEHR (low energy high resolution) collimator, was used for recording the patients’ imaging. After the injection of ^99m^Tc-DMSA, each patient underwent 3–5 planar scans (30 min-19 h), and also a single SPECT scan (2 h after injection). The time duration for each planar scan was approximately 300 s. The views of abdominal and pelvic regions including kidneys, bladder, liver, and spleen were acquisitioned so that the organs have the predominant uptake compared to the rest organs.

A triple energy window scatter correction method was used for both planar and SPECT scans. In this method, a 15% main energy window centered on the ^99m^Tc photo-peak and two 7% windows positioned on each side of the emission photo-peak. In the planar method a matrix size of 256 × 256 (pixel size = 1.75 mm) was used. For the SPECT scans, the step-and-shoot mode was utilized to acquire 40 projections over 360° with a circular orbit. The time per SPECT projection was 30 s. The SPECT images were reconstructed to a 128 × 128 matrix (resolution = 4.75 × 4.75 mm^2^ and slice thickness = 4.75 mm).

#### 2.2.2 MRI parameters

**Fig. 1:**
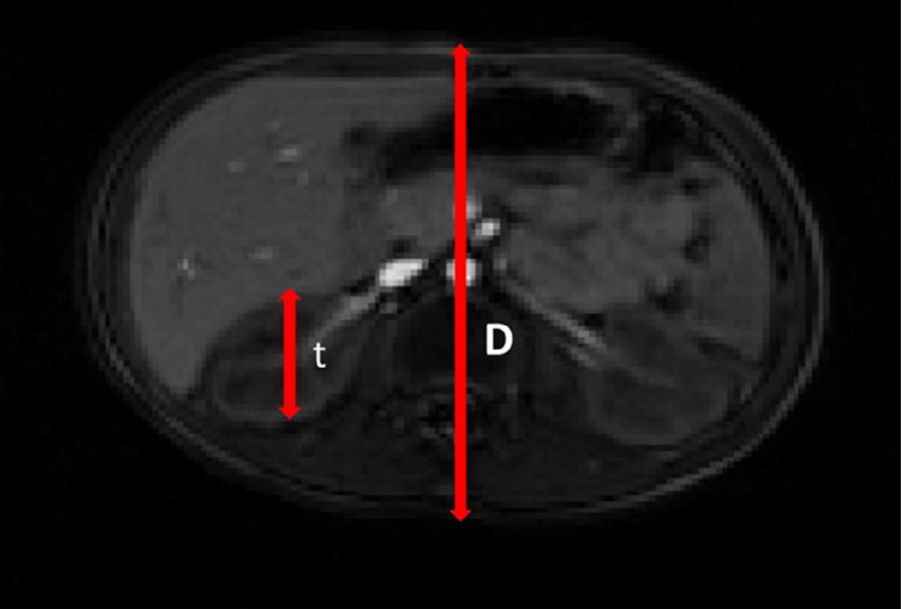
A sample of axial MRI image shown the diameter and thickness.

A Siemens Avanto MRI machine (Siemens Healthineers, Germany) with the magnetic field power of 1.5-T, was used for measuring the diameter of the patient's body and organ thicknesses for each patient (Fig. 1). The parameters of the MRI were set as repetition time (TR) =3.48 ms, echo time (TE) =1.39 ms, slice thickness 1.7 mm, and flip angle =10˚. The patient's body and organs thicknesses were measured by ITK-SNAP (version 3.6.0-RC1; http://www.itksnap.org), a free open-source segmentation software.

**Fig. 2:**
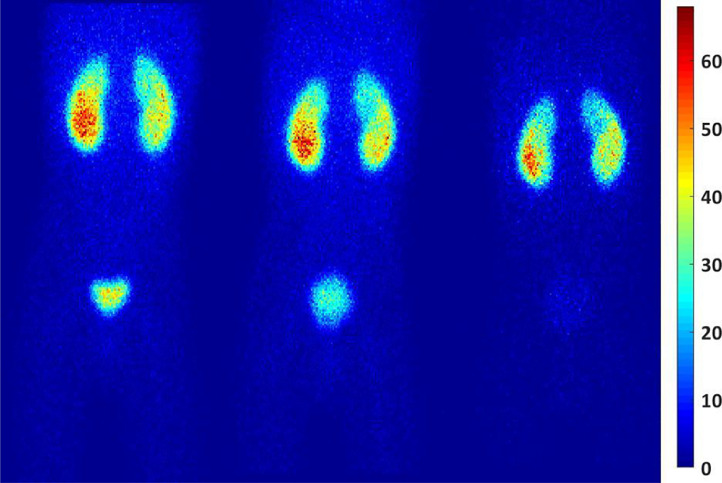
A sample of picture from patient 12 shown the biodistrubution of 99mTc-DMSA based on time. Anterior planar images acquired at 2.1 h (a), 2.86 h (b), and 19 h (c) after injection.

#### 2.2.3 Calibration factor

The calibration experiment was performed to convert the measured SPECT and planar image count rates to absolute values. To evaluate the SPECT calibration factor, a point of ^99m^Tc source (a small insulin syringe), 37 MBq, prepared and placed in air [2]. Then, the SPECT image acquired using the same parameters of the patients for the point source.

### 2.3 Activity quantification

The following equation was used to quantify the activity in each source organ A (j), MBq:

(1) $$A\left(j\right)=\;\frac{R\left(j\right)}{K.T}\;\times f$$

Where R(j) is the count rate in the drawn volume of interest, T represents the transmission factor across patient thickness and linear attenuation coefficient (0.15 cm^−1^) based on the MIRD (Medical Internal Radiation Dose) pamphlet No. 16 [4], f is the source organ self-absorption coefficient ($f=[\left(\mu_{j}d_{j}/2\right)/\sinh\left(\mu_{j}d_{j}/2\right)]$) (*μ_j_*and *d_j_*are source organ attenuation coefficient and thickness, respectively) and K is gamma camera calibration factor (cps/MBq). The differences in tissue composition and density were not included in calculations and the mean effective attenuation coefficient was used for all body organs and tissues. These values are illustrated in Table 2. In this table, the raw data of self-attenuation correction factor and thickness values for source organs and whole body (in pelvic and abdomen), also the transmission factor for whole body measured for all patients.

**Table 2:** Self-attenuation correction factor and thickness values for source organs and whole body (in pelvic and abdomen), also transmission factor for whole body measured for all patients.

Patient	Transmission factor		Self-attenuation correction factor						Thickness (cm)					
	Whole body (in pelvic region)	Whole body (in abdomen region)	Kidney	Liver	Spleen	Bladder	Whole body (in pelvic region)	Whole body (in abdomen region)	Kidney	Liver	Spleen	Bladder	Whole body (in pelvic region)	Whole body (in abdomen region)
1	0.192	0.153	0.986	0.972	0.992	0.991	0.895	0.867	3.8	5.5	3	3.1	11	12.5
2	0.165	0.142	0.986	0.968	0.992	0.990	0.877	0.857	3.9	5.9	3	3.2	12	13
3	0.192	0.138	0.984	0.972	0.995	0.992	0.895	0.854	4.2	5.5	2.2	2.9	11	13.2
4	0.237	0.149	0.988	0.975	0.992	0.992	0.919	0.863	3.6	5.2	2.9	3	9.6	12.7
5	0.165	0.156	0.990	0.977	0.993	0.989	0.877	0.869	3.3	5	2.8	3.5	12	12.4
6	0.237	0.195	0.990	0.977	0.996	0.989	0.919	0.897	3.3	5	2	3.5	9.6	10.9
7	0.091	0.067	0.982	0.942	0.992	0.985	0.795	0.750	4.4	8	3	4	16	18
8	0.223	0.149	0.985	0.977	0.993	0.990	0.912	0.863	4	5	2.8	3.2	10	12.7
9	0.173	0.160	0.989	0.976	0.994	0.988	0.882	0.873	3.4	5.1	2.7	3.6	11.7	12.2
10	0.237	0.173	0.989	0.967	0.993	0.980	0.919	0.882	3.4	6	2.5	4.7	9.6	11.7
11	0.259	0.201	0.984	0.977	0.996	0.992	0.928	0.900	4.2	5	2.8	3	9	10.7
12	0.237	0.186	0.990	0.981	0.992	0.994	0.919	0.891	3.2	4.5	2.1	2.5	9.6	11.2

### 2.4 Calculation of cumulative activity

#### 2.4.1 Time activity curves

To calculate the cumulative activity for each source organ, the hybrid planar/SPECT approach was employed. For each planar image series of patients, the ROIs were drawn around the border of the organs in the first image. Then, these ROIs were registered in the rest of image series. It is notable that the spatial distribution of activity changes during the time has decreased with this method [5,6].

A series of planar images, count rates in each ROI, were plotted against time. Then, an appropriate exponential function fit for each time-count rate curve was obtained [[1], [2], [3], 6].

Background correction was used for estimating the count rates following the equation below [3]:

(2) $$I=I^{\prime }-\left(1-\left(d_{j}\;/\;D\right)\right)\;I_{BG}$$

In this formula, the I and I′ are the background-corrected and uncorrected counts of each ROI, in that order. The d_j_ and D are the diameter of organ and patient body thickness by MR image in the axial view, and I_BG_ is the background counts. To obtain the I_BG_ value, the mean count of pixels in the background region multiplied by the number of pixels in the source organs [3].  The geometric mean of counts, (I_A_I_P_)^1/2^, was used to obtain the time-count rates curve (I_A_= anterior counts, I_P_ = posterior counts).

A sample of picture shown the biodistribution of ^99m^Tc-DMSA based on time (after injection) in Fig. 1. Also, the biodistribution in different time periods ranging from 30 min to 19 h has shown in Table 3. According to this table, the pharmacokinetic behavior of ^99m^Tc-DMSA uptake in whole-body, liver, bladder, and spleen was decreased immediately followed by a clearance phase, while, the kidneys had an opposed behavior compared to the above-mentioned organs with initial uptake phase to a maximum value. The curves were fitted with two-exponential and mono-exponential functions following their correlation coefficient values [3].

**Table 3:** Count rates (count/second) of source organs at various time after ^99m^Tc-DMSA.

Patient	Time (h)	Kidneys	liver	Spleen	bladder	Whole body
1	1.9	1104	99	13.5	153	2303
	2.5	1206	48	7.1	159	2197
	15	249	13	3	47	289
2	1.2	1123.6	205	94	45.3	2989
	2.7	1356	167	92	56	2446
	4.5	980	145	55.8	47	2100
	16.5	229	43	10	4	450
3	2.71	712.4	50.2	40.3	15.7	1316
	3.65	697.6	42.8	31.2	12.2	1249
	4.15	677.9	25.5	15	13	1173
4	0.5	315.8	59.3	49.9	14.3	1517
	2.53	709.4	51.2	42.3	15.7	1316
	3.9	655.6	44.8	29.2	14.2	1249
	7.15	355.9	15.5	10	9	1173
5	1.28	909	72	11.2	21	2300.5
	2.10	936.5	73.8	8.8	16.3	2127.1
	2.50	948.8	65	7.7	74.9	2138.1
	3.16	966.1	59.1	8.1	26.9	1954.5
	3.5	935.7	63.7	7.2	56	1992.6
6	1.13	572	126.6	18.3	134	1743
	1.63	629	111.6	12	146	1683
	2.71	633	65.7	9.8	57.1	1359
	3.33	639	42	8.1	17.5	1238
	3.7	619.6	50.3	7.3	21.4	1255
7	1.9	595.8	117	16.1	347.8	2015
	2.50	600.7	69	13.9	380	1944
	3.83	581	54.7	9.5	6.9	1254
	4.9	518	53.8	8.8	5.3	1113
8	2.2	1263.7	195	95	14.3	2819
	2.8	1312	160	88	35	2452
	4.2	987	151	59.1	8.3	2051
	17.3	219	33	9	3	436
9	1.3	435	59.9	12.8	68.9	1533
	2.4	596.5	43.2	8.6	98.6	1254
	16	153	9	3	19	168
10	1.88	1687	186.9	12.9	131.9	3306
	2.43	2105.2	106.8	15.7	138.8	3424.2
	3.00	1656.3	115.2	13.1	182.1	2927
	7.08	1183.7	87.9	7.2	22.2	1892
11	1.33	1400	124	13.3	128	2989
	2.50	1456	113	13	25	2278
	5.30	1101	65	7.6	55	2010
12	2.1	1209	95	11.8	123	2203
	2.86	1162	51	9.1	144	2152
	19	244	12	1	8	295

#### 2.4.2 Estimation of effective half lives

The effective half-lives (λ_eff_) was obtained by the planar image acquisitions. In this method [6], λ_eff_ used estimates the cumulated activity (Ã) for each organ of interest:

(3) $$∼A=\;A_{SPECT}\;\times \;\frac{e^{\lambda_{eff}t_{SPECT}}}{\lambda_{eff}}$$

In this formula, A_SPECT_ and t_SPECT_ are the activity in each source region acquired from the SPECT image and the time of the acquisition, respectively. Actually each count rate in the planar image acquisitions rescaling by each SPECT image which provides an estimate of the time-cumulated (integrated) activities [6]. The cumulated activity was calculated for the kidneys, liver, spleen, and bladder, and for the remainder of the body it was obtained by subtracting the above-mentioned organs from the whole body activity. The percentage of ^99m^Tc-DMSA uptake in source organs and the reminders are separately shown for each patient in Table 4. For obtained the percentage, the cumulative activity has calculated for source organs and whole body for each patient and then the cumulated activity of each source organ and remainder of the body divided in whole body cumulated activity.

**Table 4:** The relative percentage of ^99m^Tc-DMSA uptake calculated for each patient's organ. Also, the mean and standard deviation (SD) in source organs and the remainders are described.

	Patient													
Organs	1	2	3	4	5	6	7	8	9	10	11	12	mean	SD
Kidneys	22.6	23.7	21.2	21.9	39.9	13.4	8.0	23.9	11.5	34.2	22.9	22.4	22.1	8.8
Liver	3.6	1.8	3.7	0.9	6.7	2.7	3.9	1.8	5.2	2.6	2.9	4.4	3.3	1.6
Spleen	0.7	0.9	3.1	0.7	1.0	0.6	0.8	0.9	1.0	0.2	0.5	0.6	0.9	0.7
UB contents	2.8	1.2	0.7	0.7	4.1	6.5	20.3	1.2	3.6	5.5	2.8	2.9	4.4	5.3
Remainder	70.3	72.4	71.3	75.8	48.3	76.8	67.0	72.2	78.7	57.5	70.9	69.7	69.3	8.5

The time integrated activities were normalized to the administered activity for calculating the residence time (Table 5). Post-processing of reconstructed planar and SPECT data was performed by ITK-SNAP software.

**Table 5:** The residence time along with average (±SD) number of source organs and the remainder of the body (MBq × *h*/MBq).

Patient number	Organ Residence Time (MBq.h/MBq)				
	Kidney	Liver	Spleen	Urinary Bladder contents	Remainder of the body
1	0.69	0.05	0.04	0.05	1.01
2	2.68	0.21	0.16	0.08	3.98
3	1.90	0.17	0.16	0.03	3.03
4	2.32	0.04	0.15	0.04	3.88
5	1.93	0.16	0.01	0.10	1.15
6	0.97	0.09	0.05	0.23	2.73
7	0.39	0.09	0.04	0.49	1.58
8	2.97	0.27	0.17	0.07	4.02
9	0.31	0.07	0.03	0.05	1.06
10	3.13	0.22	0.09	0.25	2.23
11	2.06	0.14	0.07	0.13	3.17
12	0.62	0.04	0.06	0.04	0.97
Mean ± SD	1.66 ± 1.02	0.13 ± 0.08	0.08 ± 0.06	0.13 ± 0.13	2.40 ± 1.22

### 2.5 Dosimetry

The organ absorbed dose and effective dose (equivalents) were estimated for various organs of the patients (mGy/MBq) using MIRDOSE 3.1 software (Oak Ridge Institute for Science and Education, Oak Ridge, TN 37,831) shown in Table 6. The input of MIRDOSE software was residence times in source organs including kidneys, liver, spleen, and remaining body calculated in 2.4.2 section.

**Table 6:** The organ absorbed dose, effective dose (ED), and effective dose equivalents (EDE) per administered activity (mGy/MBq) for each patient using phantom based on the patient demography.

	Patient (phantom used)											
Organ dose	1 (5)	2 (5)	3 (10)	4 (5)	5 (5)	6 (5)	7 (15)	8 (5)	9 (5)	10 (5)	11 (5)	12 (5)
Adrenals	7.37E-03	2.88E-02	1.43E-02	2.52E-02	1.75E-02	1.25E-02	2.75E-03	3.13E-02	4.51E-03	2.92E-02	2.20E-02	6.82E-03
Gallbladder wall	4.91E-03	1.92E-02	9.32E-03	1.64E-02	1.04E-02	9.56E-03	2.36E-03	2.08E-02	3.74E-03	1.75E-02	1.47E-02	4.56E-03
Kidneys	8.15E-02	3.18E-01	1.58E-01	2.76E-01	2.27E-01	1.16E-01	2.39E-02	3.52E-01	3.76E-02	3.69E-01	2.44E-01	7.38E-02
Liver	4.14E-03	1.66E-02	9.00E-03	1.03E02	1.08E-02	7.44E-03	2.43E-03	1.92E-02	3.78E-03	1.68E-02	1.21E02	3.64E-03
Pancreas	5.44E-03	2.13E-02	1.12E-02	1.87E-02	1.13E-02	1.00E-02	2.48E-03	2.30E-02	3.84E-03	1.96E-02	1.59E-02	5.24E-03
Spleen	1.63E-02	6.46E-02	3.91E-02	5.93E-02	1.60E-02	2.25E-02	6.66E-03	6.93E-02	1.12E-02	4.66E-02	3.52E-02	2.13E-02
Urinary bladder wall	7.29E-03	1.56E-02	5.62E-03	1.07E-02	1.36E-02	2.99E-02	2.65E-02	1.47E-02	7.22E-03	3.24E-02	1.98E-02	6.09E-03
Gonads	2.99E-03	6.29E-03	2.88E-03	1.02E-02	4.95E-03	7.47E-03	2.69E-03	1.15E-02	2.66E-03	9.19E-03	5.35E-03	1.64E-03
ED	5.17E-03	1.93E-02	9.56E-03	1.68E-02	1.13E-02	1.06E-02	3.42E-03	2.07E-02	3.65E-03	1.97E-02	1.54E-02	4.75E-03
EDE	8.79E-03	3.36E-02	1.71E-02	2.97E-02	1.99E-02	1.55E-02	5.07E-03	3.65E-02	5.37E-03	3.48E-02	2.54E-02	8.39E-03

**Fig. 3:**
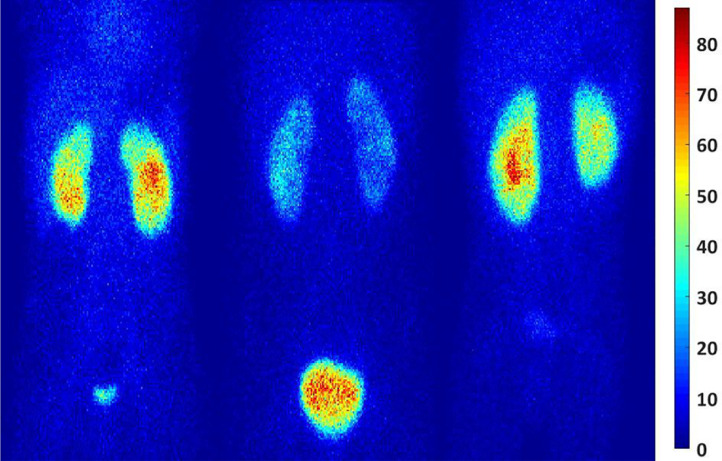
Anterior images for (a) patient 5, (b) patient 7, and (c) patient 11 at 2.50 h after injection illustrating differences in relative uptake of ^99m^Tc DMSA in the kidneys, urinary bladder contents, and liver.

The biodistribution variation at 3 patients’ accrued different functions which introduce uncertainty in absorbed dose has been shown in Fig. 3.

## Declaration of Competing Interest

The Authors declare that there is not any competing of interest regarding this article.

## Appendix Supplementary materials

Image, application 1
